# Enhancing and Not Replacing Clinical Expertise: Improving Named-Entity Recognition in Colonoscopy Reports Through Mixed Real–Synthetic Training Sources

**DOI:** 10.3390/jpm15080334

**Published:** 2025-07-30

**Authors:** Andrei-Constantin Ioanovici, Andrei-Marian Feier, Marius-Ștefan Mărușteri, Alina-Dia Trâmbițaș-Miron, Daniela-Ecaterina Dobru

**Affiliations:** 1 Department M2—Complementary Functional Sciences, Medical Informatics and Biostatistics, George Emil Palade University of Medicine, Pharmacy, Science, and Technology of Targu Mures, 540142 Targu Mures, Romania; andrei.ioanovici@umfst.ro; 2 Department M4—Clinical Sciences, Orthopedics and Traumatology I, George Emil Palade University of Medicine, Pharmacy, Science, and Technology of Targu Mures, 540139 Targu Mures, Romania; andrei.feier@umfst.ro; 3 John Snow Labs Inc., 16192 Coastal Highway, Lewes, DE 19958, USA; dia@johnsnowlabs.com; 4 Department M4—Clinical Sciences, Gastroenterology Medical VII, George Emil Palade University of Medicine, Pharmacy, Science, and Technology of Targu Mures, 540139 Targu Mures, Romania; daniela.dobru@umfst.ro

**Keywords:** synthetic data, colon polyp, prompt engineering, personalized medicine, colorectal cancer

## Abstract

**Background/Objectives**: In routine practice, colonoscopy findings are saved as unstructured free text, limiting secondary use. Accurate named-entity recognition (NER) is essential to unlock these descriptions for quality monitoring, personalized medicine and research. We compared named-entity recognition (NER) models trained on real, synthetic, and mixed data to determine whether privacy preserving synthetic reports can boost clinical information extraction. **Methods**: Three Spark NLP biLSTM CRF models were trained on (i) 100 manually annotated Romanian colonoscopy reports (ModelR), (ii) 100 prompt-generated synthetic reports (ModelS), and (iii) a 1:1 mix (ModelM). Performance was tested on 40 unseen reports (20 real, 20 synthetic) for seven entities. Micro-averaged precision, recall, and F1-score values were computed; McNemar tests with Bonferroni correction assessed pairwise differences. **Results**: ModelM outperformed single-source models (precision 0.95, recall 0.93, F1 0.94) and was significantly superior to ModelR (F1 0.70) and ModelS (F1 0.64; *p* < 0.001 for both). ModelR maintained high accuracy on real text (F1 = 0.90), but its accuracy fell when tested on synthetic data (0.47); the reverse was observed for ModelS (F1 = 0.99 synthetic, 0.33 real). McNemar χ^2^ statistics (64.6 for ModelM vs. ModelR; 147.0 for ModelM vs. ModelS) greatly exceeded the Bonferroni-adjusted significance threshold (α = 0.0167), confirming that the observed performance gains were unlikely to be due to chance. **Conclusions**: Synthetic colonoscopy descriptions are a valuable complement, but not a substitute for real annotations, while AI is helping human experts, not replacing them. Training on a balanced mix of real and synthetic data can help to obtain robust, generalizable NER models able to structure free-text colonoscopy reports, supporting large-scale, privacy-preserving colorectal cancer surveillance and personalized follow-up.

## 1. Introduction

Colorectal cancer (CRC) remains the third most common malignancy worldwide and the second leading cause of cancer death; recent surveillance data shows more than 1 million new global cases annually, with higher incidence in people under 50 years old [1].

Colonoscopy plays an important role in early detection, surveillance, and post-polypectomy follow-up of colorectal cancer (CRC), and it is part of patient surveillance after CRC resection, enabling the detection of advanced neoplasia and recurrence [2].

The increase in biomedical data for CRC offers new research possibilities but also difficulties. Traditional CRC epidemiology used cancer registries, but now advanced technology and healthcare digitization have led to an explosion of diverse data formats. These include structured registry data, unstructured clinical notes, medical imaging, and multi-omics profiles, creating rich but heterogeneous datasets. In clinical practice, large amounts of colonoscopy data are stored as unstructured free-text reports, limiting reuse for quality assurance, population-based surveillance, or even Artificial Intelligence (AI)-based decision support. Analyzing this large and varied information requires advanced methods to integrate and interpret it for easy epidemiological understanding of CRC’s causes, progression, and prevention. Combining structured and unstructured data with natural language processing (NLP) and AI generates better patient groups, improved risk assessment, and deeper clinical understandings compared to using just using one type of data alone [3].

Information extraction techniques—particularly named-entity recognition (NER), an NLP method—offer a promising solution for structuring free-text colonoscopy reports by automatically identifying clinically relevant concepts such as lesion morphology, location, and intervention details. However, the development of accurate NER models in the medical domain is heavily dependent on access to high-quality, manually annotated data. These annotated resources are time-consuming to produce, require domain expertise, and are often constrained by institutional privacy concerns and limited availability, especially in low-resource settings or for underrepresented languages such as Romanian language.

To overcome these limitations, the generation of synthetic clinical text has emerged as a potential alternative [4,5]. Synthetic data, produced through rule-based generation, templating, or generative AI, can reproduce the linguistic and semantic structure of real-world reports while avoiding patient privacy risks [6].

When validated properly, such datasets may serve as effective training material for machine learning models, reducing reliance on sensitive real-world data and accelerating the use of clinical NLP systems. In this context, synthetic colonoscopy reports represent a potentially cost-effective, scalable, and privacy-preserving resource for training NER models [7,8]. Despite their appeal, the efficacy of models trained on synthetic text has not been rigorously benchmarked against those trained on real clinical narratives. Thus, it remains an open question whether synthetically generated medical texts can provide enough fidelity and semantic diversity to support high-quality information extraction in real-world applications.

In the gastrointestinal endoscopy domain, researchers have begun applying deep learning to automatically process colonoscopy and pathology reports. For instance, one recent study introduced the first deep learning-based NER system for colonoscopy reports, highlighting the importance of this task for clinical utility (cancer risk prediction and follow-up recommendations). The model, used with domain-specific word embeddings, achieved promising results in identifying colonoscopy findings and attributes [9]. Similarly, others have developed NLP pipelines to extract detailed features from colonoscopy pathology reports with high accuracy. Benson et al. (2023) report an end-to-end pipeline that automatically identifies histopathologic polyp features (size, location, histology, etc.) from colonoscopy reports, obtaining precision as high as 98.9% and a recall of 98.0% (F1-score 98.4%) across all entities [10]. These works demonstrate that advanced NER methods can successfully capture critical clinical entities in endoscopy texts, laying the groundwork for tools that assist in automated report summarization and decision support. When data is scarce, researchers have started to leverage large language models (LLMs) to generate artificial clinical text for NER training. In a 2024 study, researchers created a synthetic annotated corpus of radiology (CT scan) reports using GPT-3.5 and used it to fine-tune a biomedical NER model. Notably, even though the LLM-generated reports cannot perfectly mimic real clinical prose, combining synthetic data with authentic data led to marked improvements in NER performance [11]. Developing effective NER in minor languages poses additional challenges due to scarce annotated data. The recent literature shows a strong interest in increasing NER models performance under low-resource conditions through means such as data augmentation. A recent 2025 study explores using synthetically generated training samples for multilingual NER in 11 low-resource languages. Their findings suggest that synthetic augmentation holds promise for improving low-resource NER [12].

In our prior research, we demonstrated the application of Spark NLP (John Snow Labs, Lewes, DE, USA) to annotate endoscopy reports and train models capable of automatically extracting relevant medical labels [13]. This approach not only enhanced the structuring of medical data but also enabled its integration with other structured datasets, paving the way for complex patient profiling.

The current study aims to further investigate the potential use of NLP and synthetic data in clinical practice and to evaluate if synthetic colonoscopy reports can improve the clinical information extraction capabilities achieved with models trained only on real medical texts. By training and comparing three NER models, one on real endoscopy reports, one on synthetic reports, and one on an equal mix, this work’s aim is to demonstrate that synthetic data provides not only a privacy-preserving, scalable alternative to real document annotations, but also substantially improves the robustness and generalization of clinical NLP models.

Quantifying the capabilities to extract clinically relevant information will clarify the feasibility of synthetic documentation for applications such as automated colorectal cancer surveillance and advanced patient profiling, reducing the dependence on manual annotation of sensitive patient data.

## 2. Materials and Methods

The real texts consisted of 100 de-identified colonoscopy reports from the Gastroenterology Department, Mureș County Clinical Hospital (Târgu Mureș, Romania), recorded between January 2021 and December 2024 and containing at least one colorectal polyp. All healthcare personal identifiers were removed before export to Generative AI Lab (John Snow Labs, DE, USA) [14].

For the synthetic texts, two gastroenterologists applied techniques of prompt engineering that reproduced the narrative style, terminology, and entity frequencies observed in the real reports. Prompts were executed within John Snow Labs Generative AI Lab, which provides access to the GPT-4 family of large language models that are optimized for clinical text generation (specifically, the model used was GPT 4o). We drafted several prompt templates, each instructing the model to produce de-identified Romanian colonoscopy reports that follow the same clinical structure but with explicit lexical variation, ultimately obtaining 100 entirely artificial but realistic colonoscopy descriptions [14]. Prompt templates and annotation guidelines are detailed in the Supplementary Materials document, Table S1 from Section S1, and annotation rules in Section S2. The same experts reviewed a subset of 50 real and 50 synthetic texts for overall plausibility and style matching. Inter-rater agreement was quantified with Cohen’s κ, and the resulting statistics are presented in the Section 3 [15].

**Figure 1:**
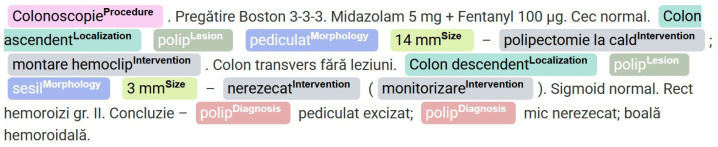
Color-coded annotation of clinical concepts in a colorectal procedure report in GenAI Lab.

Both datasets were manually annotated by two gastroenterologists for seven entities—Intervention, Lesion, Morphology, Localization, Procedure, Diagnosis, and Size—using the Generative AI Lab web interface, following a set of annotation rules, according to predefined guidelines based on the BIO (Begin-Inside-Outside) tagging scheme [16]. The annotation process via web interface is shown in Figure 1 below:

An unseen evaluation set of 20 additional real reports and 20 newly generated synthetic reports was reserved exclusively for external validation.

Three NER models were trained: ModelR (trained solely on real data), ModelS (trained only on synthetic data), and ModelM (trained on an equal mixture of real and synthetic reports). All models were implemented and trained using the No-Code Generative AI Lab platfomrm (John Snow Labs) [14,17,18]. Each model used 300-dimensional GloVe embeddings (glove_6B_300) [19], concatenated with 20-dimensional character-level embeddings for each token, as supported by the Spark NLP NER architecture [17,20].

The underlying model consisted of a bidirectional Long Short-Term Memory (biLSTM) layer for sequential feature extraction, followed by a Conditional Random Field (CRF) output layer for optimal sequence tagging, as established in prior biomedical NER studies in the literature [17,18]. Hyperparameters were set as follows: LSTM hidden size = 200, batch size = 16, learning rate = 0.001, maximum epochs = 20, and early stopping if no validation loss improvement was observed over five epochs. Default dropout and optimization settings from Spark NLP were used. All models were trained from scratch on their respective training sets using the BIO-annotated token sequences, with the validation set used for monitoring convergence.

Each model was evaluated on a reserved, unseen test set comprising 20 real and 20 synthetic reports, all manually annotated. Both token-level and span-level metrics were calculated; however, the primary evaluation metric was the micro-averaged precision, recall, and F1-score at the entity (span, exact-match) level, excluding the “O” (non-entity) class. This approach aligns with best practices established in recent clinical NLP research [21,22,23,24].

For comparative evaluation, three NER models, trained on real, synthetic, and mixed (real plus synthetic) colonoscopy reports, respectively, were assessed on a common test set consisting of 40 unseen documents (20 real and 20 synthetic). Each model’s predictions were evaluated both on the full set and separately by domain, with performance summarized using micro-averaged precision, recall, and F1-score at the entity (span-level, exact-match) and token levels.

For statistical comparison of the three NER models, McNemar’s test was applied on the combined evaluation set (40 documents: 20 real, 20 synthetic). Evaluation was conducted at the entity–span level: a span was scored as correct only when it exactly matched the gold annotation. For every pair of models, we built a 2 × 2 contingency table, counting spans that only one model, the other model, both, or neither identified correctly. The significance threshold was established at 0.05. *p* values from the three McNemar pairwise comparisons (M vs. R, M vs. S, R vs. S) were adjusted with a Bonferroni correction [25,26].

All statistical analyses, metric computations, and model-evaluation pipelines were implemented in Python 3 programming language, using the scikit-learn, statsmodels, and pandas libraries [27,28,29].

**Figure 2:**
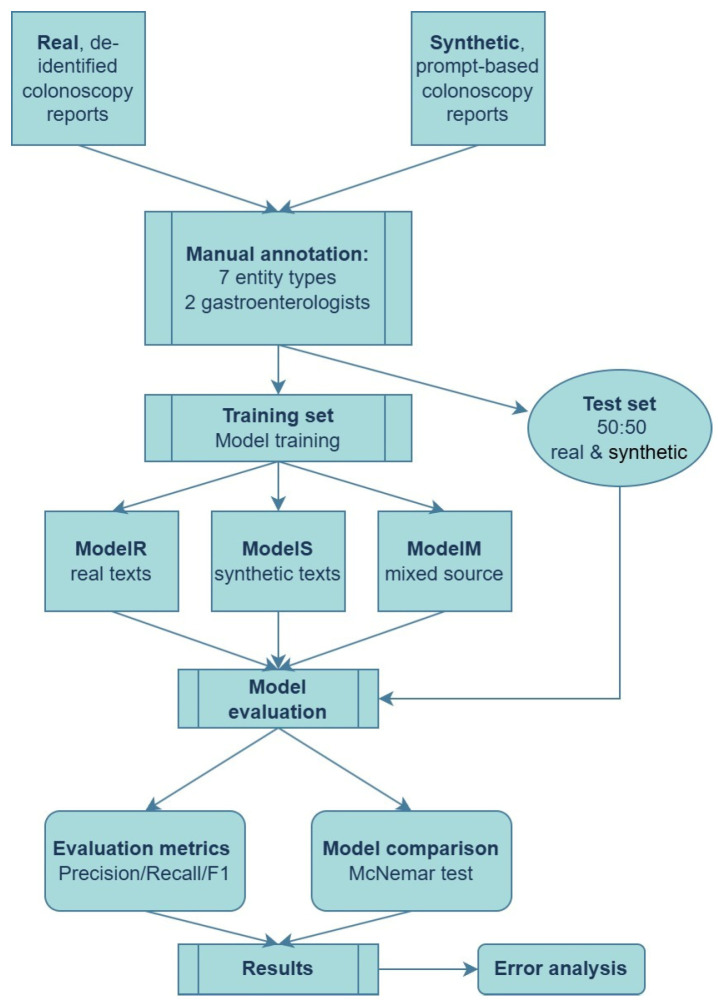
End-to-end pipeline for training and evaluating NER models on real and synthetic colonoscopy reports.

A representative flowchart of the proposed pipeline is presented in Figure 2.

## 3. Results

Inter-rater agreement was high, with Cohen’s κ = 0.85 (95% CI 0.78–0.92) for plausibility and κ = 0.81 (95% CI 0.73–0.89) for similarity, meaning that the synthetic documents closely resembled real endoscopy reports.

ModelR was trained exclusively on real colonoscopy reports and evaluated on both real and synthetic test sets. Table 1 presents ModelR’s precision, recall, and F1-score at the entity level for both domains, along with true positive, false positive, and false negative rates.

ModelS was trained only on synthetic colonoscopy reports. Table 2 summarizes its entity-level performance on real and synthetic test sets.

ModelM was trained on an equal mix of real and synthetic reports. Table 3 shows its entity-level metrics on real texts and synthetic texts, respectively.

Table 4 presents the global (combined real + synthetic) entity-level results for all models. These results reflect performance when models are evaluated across all test documents.

**Figure 3:**
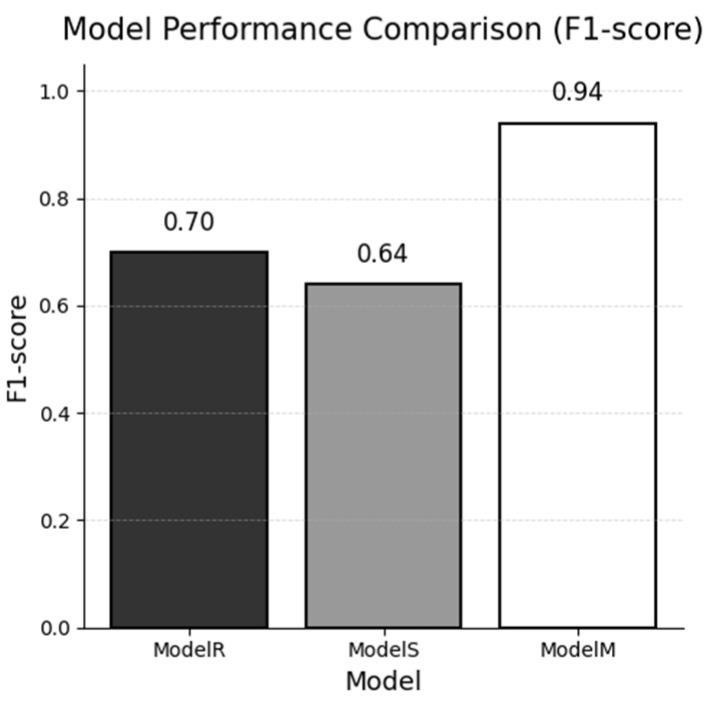
Global F1-score of each named-entity recognition model evaluated on the combined real and synthetic test sets.

Figure 3 visualizes the global F1 gap, highlighting Model M’s advantage.

Below in Table 5 are the per-entity F1-scores (span-level, “O” excluded) on the real test set.

**Figure 4:**
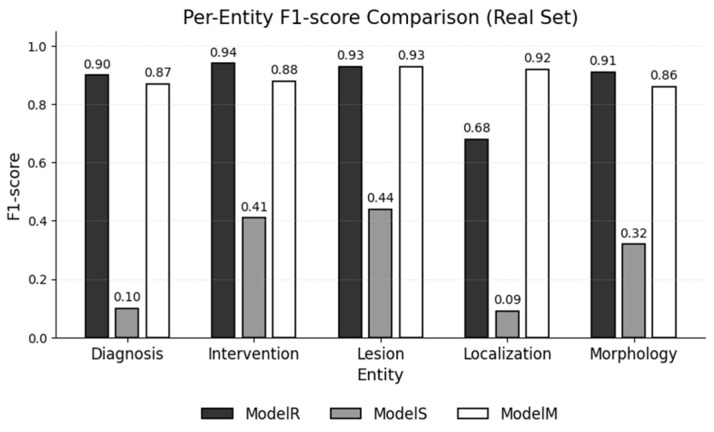
F1-scores for each entity type, as achieved by each NER model on the real test set.

Figure 4 illustrates the per-entity differences, showing that the Localization label benefits most from mixed training.

Next, we calculated the F1-scores per entity for each model, based on the synthetic data (Table 6).

The adjusted threshold for significance with Bonferroni correction was calculated to be 0.0167, and the *p* values for the McNemar tests used to compare the models are reported in Table 7 (below).

In addition to the quantitative evaluation, we performed a qualitative error analysis. These were grouped into three categories: (i) entity-type confusions (misclassifying one biomedical concept as another), (ii) omissions (false negatives), and (iii) hallucinated entities (false positives). Examples include the following:

- Type confusion by Model S, which merged the Morphology [serat] and the Lesion [polip] into a single Lesion span [“polip serat”];
- Omission by Model R, which detected the lesion [polip sesil] but missed the accompanying size [5 mm] and localization [colonul ascendent];
- Hallucinated lesion produced by Model S on real text [mucoasă], where the gold annotation contained no entities.

The mixed model detected 35 localization spans that the real model missed, which raised the recall to 0.93, with an F1-score increase from 0.68 (ModelR) to 0.92 (ModelM). Representative examples include the following:

- “Colon transvers polip pediculat 8 mm—nerezecat”
  - Gold annotations/Model M: Colon transvers—[Localization]
  - Model R: missed
- “Colon descendent polip sesil 4 mm—polipectomie la rece.”
  - Gold annotations/Model M: Colon descendent—[Localization]
  - Model R: missed
- “Sigmoid polip pediculat 19 mm—polipectomie la rece.”
  - Gold annotations/ Model M: Sigmoid—[Localization]
  - Model R: missed

Conversely, when no polyp is recorded (“Colon ascendent fără leziuni.”), both real and mixed models skip the site, respecting the annotation guidelines.

## 4. Discussion

Entity-level (span-level) evaluation is preferred for clinical NER, meaning a predicted entity is counted as correct only if the entire span and type match the gold standard exactly [30].

This exact-match criteria aligns with the BIO tagging scheme and clinical concept extraction requirements. By contrast, token-level evaluation (treating NER as word-by-word classification) is now rarely reported as a primary metric, because it can be misleading—token-level scores often look high due to the abundance of non-entity tokens. Instead, studies focus on whole-entity extraction performance. If a more forgiving metric is desired, authors sometimes include a “lenient” or overlap-based F1, which gives partial credit when a predicted span overlaps the true span [31].

For example, a study reported both exact-match and overlap-based scores, noting that exact-match F1 varied by entity and dataset, while lenient F1 was consistently high (often >0.9) when partial overlaps were allowed [32].

Globally, the mixed data trained model significantly outperformed both single-domain models, whereas the latter two both suffered marked reductions in F1 when evaluated outside their training domain. Per-entity-type F1-scores showed that the mixed model was the most consistent across all entity types. Its errors on real data consisted primarily of minor misclassification between similar entities (e.g., Lesion vs. Morphology). The model trained on synthetic data, when applied to real text, produced a high rate of false positives. The model trained on real data, conversely, showed a tendency to under-predict on synthetic reports, missing true entities.

Adding synthetic sentences expanded the lexical and syntactic coverage of “site + polyp” expressions, enabling the mixed model to capture many anatomical locations that the real-only model failed to recognize, without increasing the incidence of erroneous localization annotations. An additional contributing factor to the lower metrics of ModelS (trained exclusively on synthetic data) relates to subtle but systematic linguistic differences between the synthetic and real reports. Although the synthetic texts were reviewed by domain experts and deemed viable, qualitative inspection revealed that the synthetic reports tend to employ more standardized, consistent phrasing and a reduced range of abbreviations. Sentence structures are also generally simpler and less variable than those found in authentic clinical documentation, having greater linguistic diversity and different expressions with occasional irregularities. We believe these factors limited ModelS’s ability to recognize entities in real-world text, where greater complexity is present.

Recent studies in other clinical domains have shown that NER models trained entirely or partly on synthetic notes can approach the accuracy of models trained on real text, suggesting a viable route to overcome privacy barriers and data scarcity issues [33].

A case study on clinical entity recognition investigated training NER models on synthetic clinical notes generated by GPT-2 versus real notes. An NER model trained on a purely synthetic set of 500 notes performed similarly or slightly better than a model trained on the same number of real notes in one evaluation scenario. The authors suggest that the synthetic data contained a higher density of entities, which increased model performance. Similarly to our current research, by using a mixed training set, the authors obtained the best results, outperforming single-source-trained models [34].

In line with our study, wherein we experimented with models trained on texts in an underrepresented language (Romanian), a 2025 paper focused on a low-resource language (Estonian), generating a fully synthetic clinical text set to train NER models for medical entities. The resulting NER model achieved an F1 of 0.69 for drug name recognition and an F1 0.38 for procedure names when tested on real clinical notes, where zero real data were used for training. This demonstrates the practicality of applying clinical NLP in languages or institutions with no sharable data, by using synthetic data generation, underscoring that privacy-preserving synthetic data can effectively train usable clinical NER models [35].

A consistent finding across studies is that in-domain training data (from the same institution or context as the application data) returns the best model performance, whereas cross-domain or out-of-domain data can degrade accuracy. For example, a comparative NER study on clinical trial texts showed that, even when using the same model architecture, performance varied widely (over 20 percentage points in F1) when evaluated on different datasets from other medical institutions [36]. This highlights that models tend to overfit to site-specific text styles and terminology. In practice, NER models trained on one-source reports often see decreases in precision/recall when applied to another set, due to differences in hospital documentation conventions. Incorporating diverse training data or domain adaptation techniques can improve this. Wherever possible, external validation on a dataset from a different source is recommended to assess generalizability.

Colonoscopy documentation varies between medical facilities, and these discrepancies are prone to diminish cross-site NER accuracy. First, the report layout ranges from free-text narratives to structured templates. Furthermore, the presence of headings and checklist phrases impacts sentence boundaries and punctuation, which can shift entity spans and lead to a lower recall [37]. Second, abbreviation conventions are inconsistent: the Boston Bowel Preparation Scale may appear as “BBPS 3-3-2”, “prep 332”, or “3/3/2”, and some hospitals adopt alternative scales such as OBPS, creating words that reduce precision [38]. Third, terminological preferences differ: while many centers record polyp morphology with Paris or NICE classifications, others note only “adenom” or “polip”, and bowel-prep quality can be expressed with BBPS, OBPS or narrative adjectives [39]. Finally, regional style guides influence spelling (e.g., “colon ascendent” vs. “asc.”), adding to the lexical inconsistency. Multicenter evaluations of clinical NER consistently report 10–25-point F1 declines when models are transferred across institutions, underscoring the need for abbreviation expansion, vocabulary normalization, and domain fine-tuning before wider deployment [40].

This study used a relatively small dataset selected from a single clinical center in Romania, which may limit the generalizability of findings to broader or more diverse populations. Additionally, all annotations were performed by two experts; further validation with a larger group of annotators may improve reliability. Future studies should evaluate these models on larger and more heterogeneous datasets, including colonoscopy reports from multiple institutions and geographies, to test robustness and support wider clinical adoption. Because no inter-institutional data sharing agreement was in force during the study period, additional external validation could not be undertaken; future work will seek multicenter collaborations to assess generalizability across differing reporting conventions.

While our training set of 100 real reports is small, an important question is how the model scales with larger or differently composed datasets. Synthetic data generation offers a way to rapidly scale the training corpus, but the literature suggests there is a minimum real-data proportion needed to anchor the model’s performance. Even a small fraction of real examples can greatly enhance generalization. For example, Kamath & Vajjala (2025) demonstrate, across 11 low-resource languages, that a small amount of carefully annotated data yields better performance than a large amount of synthetic data [12]. Additionally, in a 2024 study by Ashok et al., it was found that adding just 125 human-labeled data points to a synthetic-only dataset creates a great performance increase in model metrics [41]. Similarly, in a wearable-sensor classification task, using at least 20–25% real data was necessary to reach F1-scores comparable to an all-real training scenario. When the real portion dropped to only 10%, the model’s F1 fell to 73%, even after augmenting with plentiful synthetic examples [42]. In other words, performance declines as the real-data share decreases, underscoring that models trained on extremely low real data may not perform optimally when compensating the training with synthetic data alone. The scalability of our proposed method appears promising, with the caveat that completely eliminating real data is not advisable. Our experiment with a 50/50 mix (ModelM) suggests that if more real reports beyond 100 can be obtained, even in small proportions, they could further improve the model when combined with an expanded synthetic set. Conversely, if we were to scale up the synthetic data to a 1:5 or 1:10 ratio, we would expect diminishing but still positive returns. Our future work will empirically determine the exact “tipping point” for this task, for example, evaluating 1:5 vs. 1:10 real-to-synthetic training mixes.

## 5. Conclusions

The research demonstrates that training NER models exclusively on synthetic colonoscopy reports can facilitate high performance on synthetic data, but these models do not generalize well to real clinical reports. On the other hand, models trained only on real data maintain high accuracy on similar real texts but fail to recognize entities in synthetic or out-of-domain cases. This highlights a clear limitation of relying on a single data source for clinical information extraction.

The most effective strategy is to combine both real and synthetic data for model training. Our mixed-data model achieved high, balanced performance on both real and synthetic test sets, demonstrating robust generalizability and practical utility. This mixed approach preserves patient privacy and allows for scalable model development, especially where real annotated data is scarce. Extrapolating our findings, it seems AI tools are being developed to help doctors, not replace them.

Our study is among the first to validate such approaches for Romanian-language colonoscopy reports, addressing the lack of structured data resources and advancing NLP for underrepresented languages.

Integrating synthetic data with real-world annotations increases the accuracy and reliability of structured data extraction from unstructured colonoscopy reports. This methodology supports the development of practical, privacy-conscious NLP tools for automated clinical surveillance in colorectal cancer care. The resulting structured data can enable individualized surveillance planning, contributing to a shift toward more personalized management strategies in patient care.

## Figures and Tables

**Table 1:** Evaluation metrics for ModelR on real and synthetic test sets.

Dataset	Precision	Recall	F1-Score	TP	FP	FN	Gold Count	Pred Count
Real	0.9278	0.8696	0.8978	180	14	27	207	194
Synthetic	0.5190	0.4271	0.4686	82	76	110	192	158

**Table 2:** Evaluation metrics for ModelS on real and synthetic test sets.

Dataset	Precision	Recall	F1-Score	TP	FP	FN	Gold Count	Pred Count
Real	0.3147	0.3527	0.3326	73	159	134	207	232
Synthetic	0.9948	0.9896	0.9922	190	1	2	192	191

**Table 3:** Evaluation metrics for ModelM on real and synthetic test sets.

Dataset	Precision	Recall	F1-Score	TP	FP	FN	Gold Count	Pred Count
Real	0.9059	0.8841	0.8949	183	19	24	207	202
Synthetic	0.9948	0.9896	0.9922	190	1	2	192	191

**Table 4:** Global entity-level metrics for all models.

Model	Precision	Recall	F1-Score	TP	FP	FN
ModelR	0.7443	0.6566	0.6976	262	90	137
ModelS	0.6222	0.6591	0.6404	263	160	136
ModelM	0.9491	0.9348	0.9425	373	20	26

**Table 5:** Per-entity evaluation of models on the real test set.

Entity	ModelR	ModelS	ModelM
Diagnosis	0.8966	0.1017	0.8667
Intervention	0.9444	0.4094	0.8807
Lesion	0.9259	0.4390	0.9286
Localization	0.6809	0.0909	0.9231
Morphology	0.9062	0.3226	0.8615

**Table 6:** Per-entity evaluation of models on the synthetic test set.

Entity	ModelR	ModelS	ModelM
Diagnosis	0.8500	0.8300	0.9850
Intervention	0.7600	0.7450	0.9900
Lesion	0.9000	0.8800	0.9950
Localization	0.7200	0.7100	0.9920
Morphology	0.4300	0.4200	0.9500

**Table 7:** McNemar test results when comparing models.

Comparison	b (A Correct, B Wrong)	c (A Wrong, B Correct)	chi2	*p* Value	Significance After Correction
ModelM vs. ModelR	90	9	64.646	<0.001	Yes
ModelM vs. ModelS	149	0	147.007	<0.001	Yes
ModelR vs. ModelS	150	82	19.349	<0.001	Yes

## Data Availability

The original data presented in the study are openly available in the public repository accessible at https://doi.org/10.5281/zenodo.16020981 (accessed on 17 July 2025). Prompt templates and annotation guidelines are duplicated in the Supplementary Materials. De-identified excerpts can be obtained from the corresponding author upon reasonable request and subject to an institutional data use agreement, in accordance with MDPI’s research data policy.

## Supplementary Materials

The following supporting information can be downloaded at: https://www.mdpi.com/article/10.3390/jpm15080334/s1, prompt templates, annotation guidelines, training and inference times, as well as additional training data.
