# Supplementary material for: Enhancing and Not Replacing Clinical Expertise: Improving Named-Entity Recognition in Colonoscopy Reports Through Mixed Real–Synthetic Training Sources
Source: J Pers Med. 2025 Jul 30;15(8):334. doi: 10.3390/jpm15080334 (PMC12387308; doi:10.3390/jpm15080334)
Supplement: Supplementary file 1 [file jpm-15-00334-s001.zip › jpm-3702309-supplementary.pdf]

Supplemental data

Contents

1. LLM Prompts 1

2. Annotation Guidelines: 3

3. Training and inference times 5

4. Additional training data 5

1. LLM Prompts

Prompts used to generate synthetic colonoscopy reports are depicted in Supplementary Table S1 below:

Table S1 – LLM prompts used for synthetic data generation

“You are a gastroenterologist drafting synthetic, fully de-identified Romanian colonoscopy reports for NLP training. Generate reports that follow these rules:
FORMAT
• One concise paragraph per report - no bullet lists.
• Use short, declarative sentences (8 to 15 words each).
• Mention, in order, the bowel-prep score, sedation, and every colonic segment:
Cec, colon ascendent / ascendent, colon transvers, colon descendent / descendent, sigmoid(-), rect. (If a segment is normal, say so in sentence).
• Describe any polyps with: size in mm, morphology (e.g.: sesil / plat / pediculat / serat), optional NICE/Paris pattern, intervention (e.g.: ansă rece, polipectomie la cald, hemoclipuri, biopsie, etc).
• End with one final sentence that begins with “Concluzie –” or “Diagnostic –” and lists the key findings.
- All texts should have at least one polyp!
LANGUAGE & STYLE
• Romanian medical terminology only.
• Correct accents and grammar (e.g., “diverticuloză sigmoidiană”, “sesil”, “apendicular”).
• Vary phrasing: alternate “colon ascendent” / “ascendent”, “pregătire bună” / “pregătire Boston 3-3-3”, etc.
• Use realistic Boston scores (e.g., 3-3-2, 2-2-2, 3-3-3).
• No PHI or real patient data; everything must be synthetic.
EXAMPLES (for style guidance only – do not copy)

▪ “Endoscopie digestivă inferioară -- Pregatire medie. Se avanseaza pana la valva ileocecala. Langa valva ileocecala, polip sesil de aproximativ 1 cm cu structura NBI de adenom. Nu se poate efectua polipectomia din cauza prezentei materiilor fecale in cec. Ascendent, transvers – normal. La 60 cm de MA – polip de aproximativ 2,5 cm, pedicul gros aproximativ 1 cm, polipectomie cu sangerare imediata. Se monteaza 2 hemoclipuri, se injecteaza Adrenalina 1-10.000 3x2 ml. Sangerarea continua. Concluzii- Polip colonic excizat endoscopic.”

▪ “Colonoscopie [DATE]- [DOCTOR]- Se efectueaza analgosedare cu Propofol 120 mg. Se avanseaza pana la 30 cm de MA, unde se deceleaza polip mare de aproximativ 3 cm, polilobat, cu pedicul lung cu grosime de aproximativ 1 cm NBI NICE II cu zone NICE III. Se efectueaza polipectomie cu ansa, cu montare de doua hemoclipuri la baza pediculului, cu recuperarea polipului. Rect cu dilatatii hemoridale. Concluzii- polip colonic ectomizat endoscopic. Boala hemoroidala.”

You are an expert gastroenterologist creating anonymous, Romanian-language colonoscopy narratives for an NLP dataset.

STRICT SPECS

1. One dense paragraph per case — no lists or line breaks inside.
2. Each paragraph must open with “Colonoscopie”, “Colonoscopie totală”, “Endoscopie digestivă inferioară” or “Rectosigmoidoscopie”.
3. Immediately state bowel-prep quality (either a Boston 3-3-3 / 3-3-2 / 2-2-2 score or an adjective like “pregătire bună”).
4. Give sedation drug & dose (multiples of 5 mg).
5. Describe, in anatomic order, every segment: cec, colon ascendent, colon transvers, colon descendent, sigmoid, rect.
   • If normal, ≤ 5-word note (ex. “Colon transvers fără leziuni”).
6. Polyps / lesions (≥ 1 per report):
   • size mm • morphology (sesil / pediculat / plat / serat/ etc.)
   • optional NICE / Paris code
   • intervention if removed (ansă rece, polipectomie la cald, hemoclip, biopsie).
   • ~15 % of the time the polyps may not be removed (add plausible medical reason) “nerezecat (trombocitopenie / pregătire slabă / diametru mare)”.
   • Tumoral findings allowed: “formațiune vegetantă/tumorală” with biopsies, not resected.
7. End with a single concluding sentence beginning “Concluzie –” or “Diagnostic –” summarising key findings.

STYLE & TERMINOLOGY

• Romanian medical lexicon, diacritics included.
• Short declarative sentences (8-15 words).
• Vary wording (“ascendent” vs “colon ascendent”, “sigmoid normal” vs “sigmoid fără leziuni”).
• No PHI, no unrequested pathologies (avoid “colită actinică” etc.).
• Every report must contain at least one polyp or tumoral lesion.

Role: senior endoscopist. Objective: produce synthetic Romanian colonoscopy paragraphs for NLP.

GUIDELINES
1. Paragraph only, no numbering.
2. Header word: “Colonoscopie”, “Colonoscopie totală”, “Endoscopie digestivă inferioară” or “Rectosigmoidoscopie”.
3. Bowel prep:
• Colonoscopie/Endoscopie digestivă inferioară: give Boston (3-3-3, 3-3-2, 2-2-2).
• Rectosigmoidoscopie: give qualitative phrase (“pregătire adecvată”, “pregătire necorespunzătoare”).
4. Sedation: Propofol / Midazolam, rounded dose (180-240 mg).
5. Sequence each anatomical segment; mark normal ones briefly.
6. Lesions:
  – Minimum one polyp or tumoral mass per report.
  – Polyp attributes: size mm, morphology (sesil / pediculat / plat / serat), intervention (ansă rece, polipectomie la cald, clip, biopsie).
  – Tumor example: “colon ascendent formațiune ulcerată 35 mm – biopsii, nerezecată”.
7. Close with “Concluzie –” summarising findings.

STYLE & RULES
• Romanian with diacritics, 7-20-word sentences.
• Alternate wording for segment names and prep descriptions.
• No real patient identifiers;
• Cases may include other pathologies (“hemoroizi” or “diverticuloză”) for variety.

2. Annotation Guidelines:

Annotation Guidelines for the Romanian Colonoscopy-NER Corpus

Scope and objective

These guidelines define how seven clinically relevant concepts must be annotated in Romanian colonoscopy reports so that a high-precision NER model can structure free-text endoscopy documentation for quality-of-care surveillance and research.

General annotation principles

Tagging schemeBIO (B-LABEL, I-LABEL, O).

Span minimality - Mark the shortest phrase that still conveys the concept; trim leading/trailing blanks and punctuation.

Multiple mentions - Tag every occurrence, even if repeated or negated.

Negation & temporality - We do not assign separate assertion labels; a negated phrase is simply not annotated.

Ambiguity rule - If a phrase could belong to two labels, choose the more specific medical concept (e.g., “polipectomie la cald” as [Intervention]).

Entity definitions and examples

Table S2 – Entity definitions

Entity	Definition	Include	Exclude	Example
Lesion	Any polyp or mucosal abnormality addressed during the procedure.	polip, adenom, neoformațiune	Pure morphology words; general anatomy	“polip pediculat”
Morphology	Shape or surface descriptors of a lesion.	sesil, plat, pediculat, serat, Paris 0-IIa	Size, location, intervention	“polip serat de 6 mm”
Size	Numeric dimension of a lesion (with or without unit).	5 mm, 0,8 cm, cca 2 cm	Numeric values attributed to different concepts	“leziune de 12 mm”
Localization	Colonic segment or distance marker where the lesion/intervention occurs.	cec, colon ascendent, 40 cm de AO	Terms without anatomical value (“proximal”)	“polip în colon transvers”
Intervention	Immediate therapeutic/diagnostic act applied to the lesion.	Bio-exereză, ansă rece, polipectomie la cald, biopsie, hemoclipuri	Sedation, bowel prep	“s-a efectuat polipectomie la cald”
Procedure	The overall endoscopic examination. Usually once per report.	Colonoscopie totală, Endoscopie digestivă inferioară	Section headings, conclusions	“Colonoscopie sub analgosedare”
Diagnosis	Final clinical statement of findings, typically in the concluding sentence.	adenom tubulo-vilos, diverticuloză sigmoidiană	Pure morphology terms	“Diagnostic – adenom villos”

Workflow

- Read the report to understand context.

- Highlight entities following the order in the table to reduce overlap errors.
- Verify boundaries with the tool's preview.
- Mark Ready for QA and proceed to the next report.

Quality assurance

Dual annotation with reconciliation; target Cohen's $\kappa \geq 0.80$.

During adjudication check for common slips and mark for discussion.

3. Training and inference times

The table below presents the times for each model for training and inference:

Table S3 – Training and inference times for each model

Model	Training Time	Inference Time
ModelM	23 minutes 31 seconds	2.7 ms
ModelR	19 minutes 43 seconds	1.11 ms
ModelS	19 minutes 21 seconds	1.10 ms

4. Additional training data

To compare entity-level performance across both domains at a glance, Figure 1 presents a heat map of F1-scores:

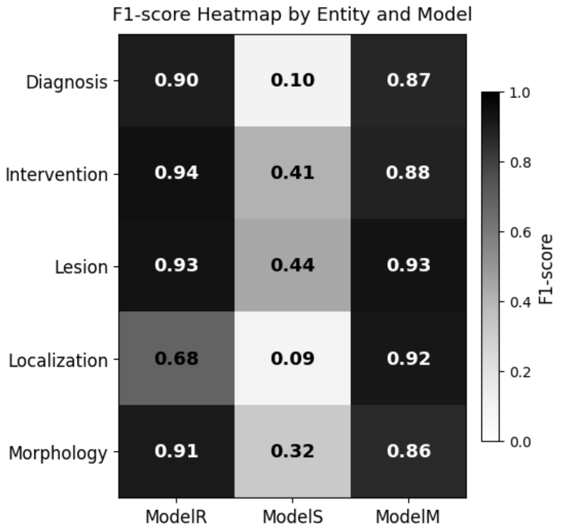

Figure S1 – F1 heatmap by model
